# The benefits of HOPE are fully certain?

**DOI:** 10.1097/JS9.0000000000000853

**Published:** 2023-10-26

**Authors:** Yu Xiao

**Affiliations:** The People’s Hospital of Longhua, Shenzhen City, Guangdong Province, People’s Republic of China

*Dear Editor*,

Recently, Tang and colleagues carried out an interesting meta-analysis regarding the prognosis of the Hypothermic Oxygenated Machine Perfusion (HOPE) in liver transplantation^1^. In this meta-analysis^1^, authors performed the first-time literature retrieval on 15 June 2023, and then did the updated literature retrieval on 12 August 2023. Since the authors had done the updated literature retrieval, they were able to include the latest relevant trial of NCT04812054 (ClinicalTrials.gov Identifier)^2^. Doing such a thing is essential and appropriate. After the authors conducted meta-analyses on 14 clinical outcomes, that is, major postoperative complications, primary nonfunction, reperfusion syndrome, hepatic artery thrombosis, renal replacement therapy, length of hospital stay, 1-year recipient death, intensive care unit stay after liver transplantation, biliary complications, non-anastomotic biliary strictures (NAS), early allograft dysfunction (EAD), acute rejection, retransplantation rate, and 1-year graft loss rate; they concluded: Compared with static cold storage (SCS), HOPE can reduce the incidence of biliary complications, NAS, EAD, acute rejection, retransplantation, and 1-year graft loss. Moreover, the authors stated in their conclusions that HOPE versus SCS can contribute to minimizing complications and enhancing graft survival in liver transplantation. These statements seem to be fully certain. However, there are several points that probably affect the certainty of the findings in this meta-analysis^1^.

First, the authors assessed a total of 14 clinical outcomes in their meta-analysis^1^ but failed to distinguish primary outcomes from secondary outcomes. In this case, the multiple testing issue should be considered in the study. Since the total number of the outcomes evaluated in this meta-analysis is 14, the significance threshold after adjusting multiple comparisons should be calculated by 0.05 ÷ 14=0.00357, according to the Bonferroni correction method^3^. According to this adjusted threshold for statistical significance, in this meta-analysis, the significant findings regarding many outcomes, such as total biliary complications (*P*=0.004), acute rejection (*P*=0.03), and retransplantation (*P*=0.03), would not be able to reach statistical significance. This could obviously change the conclusions of this study.

Second, sensitivity analyses in this study^1^ revealed that the results of meta-analyses were not robust in terms of some outcomes, such as retransplantation, hospital stay, and acute rejection; however, the ‘Discussion’ section of this article^1^ did not give a relevant explanation. Moreover, this point was not taken into consideration when the conclusions were drawn.

Last, in this meta-analysis^1^, the authors did not perform the quality assessment for evidence. Since the authors carried out this meta-analysis by simultaneously incorporating both randomized trials and observational studies, it would be difficult to assess the strength of evidence (SOE). This is because evidence deriving from randomized trials typically initially starts with a provisional grade of high SOE, whereas evidence deriving from observational studies initially starts with a provisional grade of low SOE^4^. Moreover, authors did not perform detection of publication bias, which is essential for the assessment of SOE.

## Ethical approval

Ethical approval was not required because this article does not contain any studies with human subjects or animals performed by any of the authors.

## Consent

Informed consent was not required because this article does not contain any studies with human subjects performed by any of the authors.

## Sources of funding

This work did not receive any type of funding.

## Author contribution

Y.X.: study concept and writing the paper.

## Conflicts of interest disclosure

The author declares that there are no conflicts of interest.

## Research registration unique identifying number (UIN)

This paper is a Letter to the Editor and registration is not needed.

## Guarantor

This paper is a Letter to the Editor and the guarantor is not needed.

## Data availability statement

No datasets were generated or analyzed during the current study.

## Provenance and peer review

This paper was not invited.
